# *In Vivo* and *In Vitro* Evidence for Placental DNA Damage in Preeclampsia

**DOI:** 10.1371/journal.pone.0086791

**Published:** 2014-01-22

**Authors:** Serkalem Tadesse, Dawit Kidane, Seth Guller, Tianmeng Luo, Nicholas G. Norwitz, Felice Arcuri, Paolo Toti, Errol R. Norwitz

**Affiliations:** 1 Department of Obstetrics & Gynecology, Tufts Medical Center, Boston, Massachusetts, United States of America; 2 Mother Infant Research Institute (MIRI), Tufts University School of Medicine, Boston, Massachusetts, United States of America; 3 Department of Therapeutic Radiology and Genetics, Yale University School of Medicine, New Haven, Connecticut, United States of America; 4 Department of Obstetrics, Gynecology & Reproductive Sciences, Yale University School of Medicine, New Haven, Connecticut, United States of America; 5 Department of Molecular and Developmental Medicine, University of Siena, Siena, Italy; 6 Department of Medical Biotechnologies, University of Siena, Siena, Italy; Chinese Academy of Sciences, China

## Abstract

Preeclampsia (PE) is an idiopathic multisystem disease affecting 5–7% of pregnant women. Placental oxidative stress is a characteristic feature of PE and occurs when the production of reactive oxygen species (ROS) within the placenta overwhelms the intrinsic anti-oxidant defenses. We hypothesize that excessive oxidative DNA damage at the fetal-maternal interface coupled with a defective DNA damage/repair response is causally related to PE. Here we demonstrate that γH2AX (a sensitive marker of DNA damage) is expressed in the maternal decidua but not trophoblast of normal placentas, and that expression is significantly higher in PE placental tissues *in vivo*. Using primary *in vitro* cultures of maternal decidual stromal cells (DSCs) and fetal cytotrophoblast cells (CTs), we show an increase in γH2AX foci in DSCs cultured with vs without H_2_O_2_ (70.6% vs 11.6%; *P*<0.0001) or under hypoxia-reperfusion vs normoxia (20- vs 3-fold; *P* = 0.01); no foci were seen in CTs. We further demonstrate that Base Excision Repair (BER) intermediates are significantly increased in DSCs (not CTs) under these same conditions. Our data show that DNA damage is significantly more common in PE placentas, and that this DNA damage is localized to the maternal and not fetal side of the placenta. CTs may be selectively resistant to DNA damage in an effort to protect the fetus.

## Introduction

Preeclampsia (PE) is an idiopathic multisystem disease affecting 5–7% of pregnant women. It is characterized clinically by new-onset hypertension and proteinuria after 20 weeks of gestation [1]. While the exact pathophysiology of PE remains unclear, there is evidence to suggest that such processes as abnormal trophoblast invasion, placental ischemia, and an imbalance in circulating vasoactive regulatory factors are implicated leading to generalized endothelial injury and vasospasm [2]. Excessive placental oxidative stress appears to be an important inciting event [3], [4].

The high oxygen demand of the mother and fetus increases oxidative metabolism and boosts free radical generation during pregnancy [5]. Mitochondrial superoxide anion (O_2_^−^) production is a critical source of oxidative stress within the placenta, and contributes to the overall increase in maternal and placental lipid peroxidation seen in pregnancy [6]–[8]. In healthy pregnancies, a balance is maintained between lipid peroxides and anti-oxidative processes. In contrast, PE pregnancies are characterized by an imbalance in these processes leading to an increase in oxidative stress [6]–[8]. Oxygen concentrations at the fetal-maternal interface fluctuate during pregnancy as a consequence of the vascular remodeling within the tissues of the uterus [9]. Infection/inflammation, intense tissue remodeling, and changes in vascular perfusion generate reactive oxygen species (ROS)–including O_2_^−^, hydroxyl radical, and hydrogen peroxide (H_2_O_2_)–all of which are capable of damaging nucleic acids, proteins, and lipids if levels of these ROS overwhelm the intracellular anti-oxidative defenses [10].

DNA damage induced by ROS is repaired by a series of specific and non-specific repair mechanisms [11], [12]. Base Excision Repair (BER) is one of the major DNA repair pathways in eukaryotic cells. It is initiated by DNA glycosylases, enzymes that recognize and cleave the damaged bases thereby creating sites along the DNA strand that are temporarily devoid of a base, known as apurinic/apyrimidinic (AP) sites [11]. These AP sites are then further processed by DNA glycosylases family members with AP-lyase or AP-endonuclease activity (such as APE1), which fill in the single nucleotide gap and seal over the hole in the DNA strand to complete the repair reaction [13]. Importantly, until they are fully repaired, these AP sites are both cytotoxic and mutagenic [14]–[16]. Therefore, although these DNA repair mechanisms are critical for the maintenance of genomic stability, they generate intermediates that can exacerbate DNA damage. High numbers of AP sites can therefore overwhelm the BER machinery leading to further DNA damage [17], [18].

Traditional concepts hold that ROS-mediated DNA damage occurs randomly with a tissue, which then triggers activation of the BER pathway to remove and replace the damaged bases to minimize their potentially pathogenic consequences. However, emerging evidence suggests that ROS-mediated DNA damage is not random. Rather it may be localized to one or more specific cell populations within any given tissue. We hypothesize that this may be true also in the placenta. Exactly how the balance between ROS-induced DNA damage and repair is maintained at the fetal-maternal interface is not known, but the association between high levels of circulating markers of DNA damage and PE has been previously reported [19]. In this study, we hypothesize that ROS-mediated DNA damage may lead to targeted injury to specific cell populations at the fetal-maternal interface, which may have important implications for the pathogenesis of PE.

## Materials and Methods

### Reagents

Cell culture media (DMEM/F12), estradiol (E_2_), medroxyprogesterone acetate (MPA), HEPES, BSA, FeSO_4_, ZnSO_4_, CuSO_4_, ascorbic acid were purchased from Sigma-Aldrich (St. Louis, MO). Penicillin and streptomycin, L-Glutamine, sodium pyruvate, and trypsin were purchased from Invitrogen (Burlington, Canada). Collagenase-DNase and protease inhibitors (complete cocktail) for tissue digestion were from Roche (Montreal, Canada). Reagents for sodium dodecyl sulfate-polyacrylamide gels (SDS-PAGE) were supplied by Bio-Rad (Bio-Rad Laboratories, Hercules, CA). Enhanced chemiluminescence kit was purchased from Thermo Fisher Scientific, (Rockford, IL). Insulin/transferrin/selenium and epidermal growth factor were purchased from BD Biosciences (San Jose, CA). Fetal bovine serum (FBS) and charcoal-stripped calf serum (SCS) was purchased from Gemini Biosciences (West Sacramento, CA). Primary antibodies included: anti-histone H2A.X (D17A3) XP® rabbit mAb (#7631) and α-tubulin (#2144) from Cell Signaling (Danvers, MA); anti-vimentin (Clone V9) and anti-cytokeratin-7 (Clone OV-TL 12/30) from DakoCytomation (Carpinteria, CA); and anti-CD45 mAb, anti-CD9 mAb, and isotype-matched control antibodies from R&D Systems (Minneapolis, MN). Goat anti-mouse IgG immunomagnetic beads (Dynabeads) were purchased from Dynal (Oslo, Norway). Secondary antibodies such as horseradish peroxidase (HRP)-conjugated, FITC-conjugated and biotin-conjugated anti-mouse and anti-rabbit antibodies were purchased from Jackson ImmunoResearch Laboratories (West Grove, PA). For flow cytometry, BD Biosciences supplied FITC-conjugated goat anti-mouse IgG (Bedford, MA). For immunohistochemistry (IHC) and immunocytochemistry (ICC), avidin-blocking kit, avidin-biotin-peroxidase kit with 3,3-diaminobenzidine tetrahydrochloride dehydrate (DAB) and Vectashield mounting medium with DAPI (H-1500) were purchased from Vector Laboratories (Burlingame, CA). For RT-qPCR, pre-validated human primers were from TaqMan Assay-On-Demand (Applied Biosystems, Foster City, CA), including γH2AX (Hs00266783_s1) and 18S (Hs99999901_s1). DNA_ZOL_, Trizol, SuperScript II reverse transcriptase, and oligo dT were purchased from Invitrogen (Carlsbad, CA).

### Subjects and Specimens

Tissues for IHC were collected from women with or without PE and prepared as previously described [20], [21]. In all cases, intra-amniotic infection (IAI) was excluded on the basis of clinical criteria (absence of fever, maternal and/or fetal tachycardia, uterine tenderness, and foul lochia) as well as routine laboratory investigations (white cell count) and the absence of chorioamnionitis at the time of histological examination by a single placental pathologist (P.T.) who was blinded to the clinical status of the women. Collection of tissues for this study was approved by the Human Investigations Committee (Institutional Review Board) of Yale University School of Medicine in New Haven, CT, and the Institutional Review Board of the University of Siena in Siena, Italy. Written patient consent was waived by the approving Institutional Review Boards.

### Immunohistochemical Studies

Serial sections (4 µm) of paraffin-embedded tissues were cut, de-paraffinized, rehydrated, and washed in Tris-buffered saline (20 mmol/L Tris-HCl, 150 mmol/L NaCl, pH 7.6). Antigen retrieval was performed in citrate buffer (10 mmol/L, pH 6.0) under slow boil for 8 min. Endogenous peroxidase activity was quenched by incubating the slides in 3% H_2_O_2_ for 15 min. The slides were then incubated in 10% normal donkey serum diluted in PBS/0.1% Tween-20 with an avidin blocking kit for 1 h at room temperature (RT) in a humidifying chamber. After blotting off excess serum, slides were incubated overnight at 4°C with primary antibodies prepared in PBS/0.1% Tween-20 and biotin blocking buffer. Thereafter, slides were washed and incubated with secondary antibody for 30 min at RT. After further washing, the antigen-antibody complex was detected using an avidin-biotin-peroxidase kit with DAB as the chromogen. Slides were counterstained with hematoxylin (Sigma, St Louis, MO) and mounted. Negative controls for each section were prepared by substituting pre-immune serum for the corresponding pre-immune antibody.

### H-Score

γH2AX immunostaining intensity was evaluated semi-quantitatively using AxioVision and the corresponding digital image processing software (Carl Zeiss Micro Imaging, Thornwood, NY) as previously described [20], [21] according to the following scale: 0 (no staining), 1 (weak but detectable staining), 2 (moderate staining), or 3 (intense staining). In brief, the H-SCORE was determined by calculating the sum of the percentage of cells that stain at each intensity scale and multiplying that value by the weighted intensity scale using the following formula: HSCORE = Σ*_i_* i X P*_i_* where “*i”* represents an intensity score and “P*_i_*” is its corresponding percentage of cells. In each slide, 5 fields and at least 100 cells per field were evaluated under a light microscope (×200 magnification). Scoring was performed by a single investigator (S.T.) who was blinded to the clinical status of the women. Results are reported as mean±SEM from a minimum of 5 separate readings from 3 separate tissue sections.

### Isolation and Purification of CTs

Primary cultures of CTs were isolated from human term placentas and cultured using established methodology [20], [21]. Briefly, villous placental tissue was minced, sequentially digested, and filtered through a stainless steel sieve. The filtrate was centrifuged and resuspended in FBS-containing medium to stop the digestion. The supernatant was then separated on a 4-layered discontinuous Percoll gradient, and the enriched cell fraction subjected to immunopurification by negative selection using incubations with antibodies mentioned above conjugated to immunomagnetic beads. Thereafter, contaminating immune and fibroblast cells were magnetically separated from the negative cell fraction, and the unbound cells were collected, washed, and cultured in a 95% air/5% CO_2_ incubator at 37°C in DMEM supplemented with 10% FBS. The purified term trophoblast cell populations were characterized by flow cytometry as previously described [22]. The immunomagnetic microsphere purification resulted in CTs that contained <1% contaminating vimentin-positive fibroblasts and <0.4% contaminating CD45-positive immune cells (data not shown).

### Isolation of DSCs

Term DSCs were prepared as previously described [21], [23]–[25]. In brief, decidual tissues were scraped from the maternal surface of the chorion, minced, and digested in Ham’s F-10 medium containing 10% SCS and 25 mg/mL Collagenase-DNase for 75 min. The digested tissue was passed through a 23 gauge needle to dissociate remaining cell clusters, purified on a Percoll gradient, grown to confluence in a 95% air/5% CO_2_ incubator at 37°C, and passaged until ICC revealed that DSCs were more than 99% pure (vimentin-positive) and free of contaminating CD45-positive and cytokeratin-positive cells (data not shown).

### Experimental Incubations for CTs and DSCs

A total of 7×10^6^ CTs were plated in T25 flasks (Falcon) and cultured at 37°C in a 95% air/5% CO_2_ incubator. For decidual experiments, 5×10^5^ DSCs were also plated in T25 and grown to confluency. They were then treated with 10^−8 ^mol/L estradiol (E_2_) and 10^−7 ^mol/L medroxyprogesterone acetate (MPA) for 7 days [21], [23]–[25]. Thereafter, DSC cultures were washed twice with HBSS to remove residual serum and switched to a serum-free defined medium containing insulin/transferrin/selenium, 5 mmol/L FeSO_4_, 0.5 mmol/L ZnSO_4_, 1 nmol/L CuSO_4_, 50 mg/L ascorbic acid, and 50 ng/mL epidermal growth factor. For the hypoxia/reperfusion (HPX/R) experiments, cells were cultured under <2% O_2_ for 24 h and then with fresh media equilibrated at 21% O_2_/5% CO_2_ for an additional 6 h. Cells cultured at 21% O_2_/5% CO_2_ for 24–36 h will serve as a normoxic (NMX) control [26], [27]. In a separate set of experiments, cells were cultured under NMX for 1 h with or without H_2_O_2_ (100 µM) to generate excess ROS. These experimental paradigms were chosen based on prior dose-response and time-course experiments [21], [23]–[25].

### Western Blot

Protein was extracted from whole cell lysates from each treatment group as previously described [28], [29]. Equal amounts of protein (30 µg) were then loaded on precast 4%–15% SDS-PAGE gels under reducing conditions and transferred to a nitrocellulose membrane. Membranes were incubated overnight with primary antibody in 5% milk in Tris-buffered saline with Tween-20 at 4°C and then secondary antibody coupled with HRP (1∶5000–10000) in 5% milk at RT for 1 h. Positive signals were detected by an enhanced chemiluminescence kit according to the manufacturer instruction. The density of the band was measured using NIH Image J software, and the relative signals were quantified as the ratio of γH2AX phosphorylated to α-tubulin.

### Nuclear Protein Localization in CTs and DSCs Using Confocal Microscopy

To localize γH2AX staining, CTs and DSCs were grown on chamber slides and fixed with methanol:acetic acid (3∶1 ratio) for 15 min at −20°C. Cells were then permeabilized in PBS containing 0.5% Triton X-100 for 10 min at RT, incubated with anti-γH2AX antibody for 1 h at RT, and staining detected with a secondary FITC-conjugated antibody. Antibody dilutions and washes were performed in PBS. Finally, chamber slides were mounted in Vectashield mounting medium with DAPI and visualized using a Zeiss LSM 510 META confocal microscope processed by Zeiss LSM software (Carl Zeiss, Oberlocken, Germany).

### DNA Damage Analysis by Detection of AP Sites

To determine if AP sites represent the major class of DNA damage induced by H_2_O_2_ treatment, we measured the number of DNA AP sites per nucleotide in cultured DSCs and CTs with a previously described ELISA-like assay that utilizes an aldehyde reactive probe (ARP) and has been shown not to introduce additional AP sites [30]–[32]. In brief, term CTs and DSCs treated with/without H_2_O_2_ were washed three times with PBS and genomic DNA prepared using DNA_ZOL_ reagents. The DNA was then immobilized on a 96-well plate with DNA binding solution, incubated with streptavidin-conjugated HRP, and rinsed with washing buffer. After adding 100 µL of substrate solution to each well and incubating the 96-well plate at 37°C for 1 h as recommended by the manufacturer, the enzymatic activity of HRP was detected calorimetrically by measuring absorbance at 450 nm. The number of AP sites was calculated based upon a standard curve generated using ARP standard DNA solutions according to the manufacturer’s protocol (Abcam, Cambridge, MA).

### Statistical Analysis

Data were evaluated using GraphPad (GraphPad Software Inc., San Diego, CA) and SigmaStat (Jandel Scientific Corp., San Rafael, CA). All data sets were subjected to normality testing. H-SCORE data sets were analyzed using the Holm-Sidak test for both pair-wise comparisons and comparisons versus control groups. These data are reported as mean±SEM. Normally distributed results were analyzed by ANOVA and Student's *t*-test and expressed as mean±SEM? *In vitro* assays were performed in triplicate and experiments were performed a minimum of three times to verify results. Statistical differences are reported.

## Results

### Oxidative DNA Damage is Enhanced in PE Placentas *in vivo*

**Figure 1:**
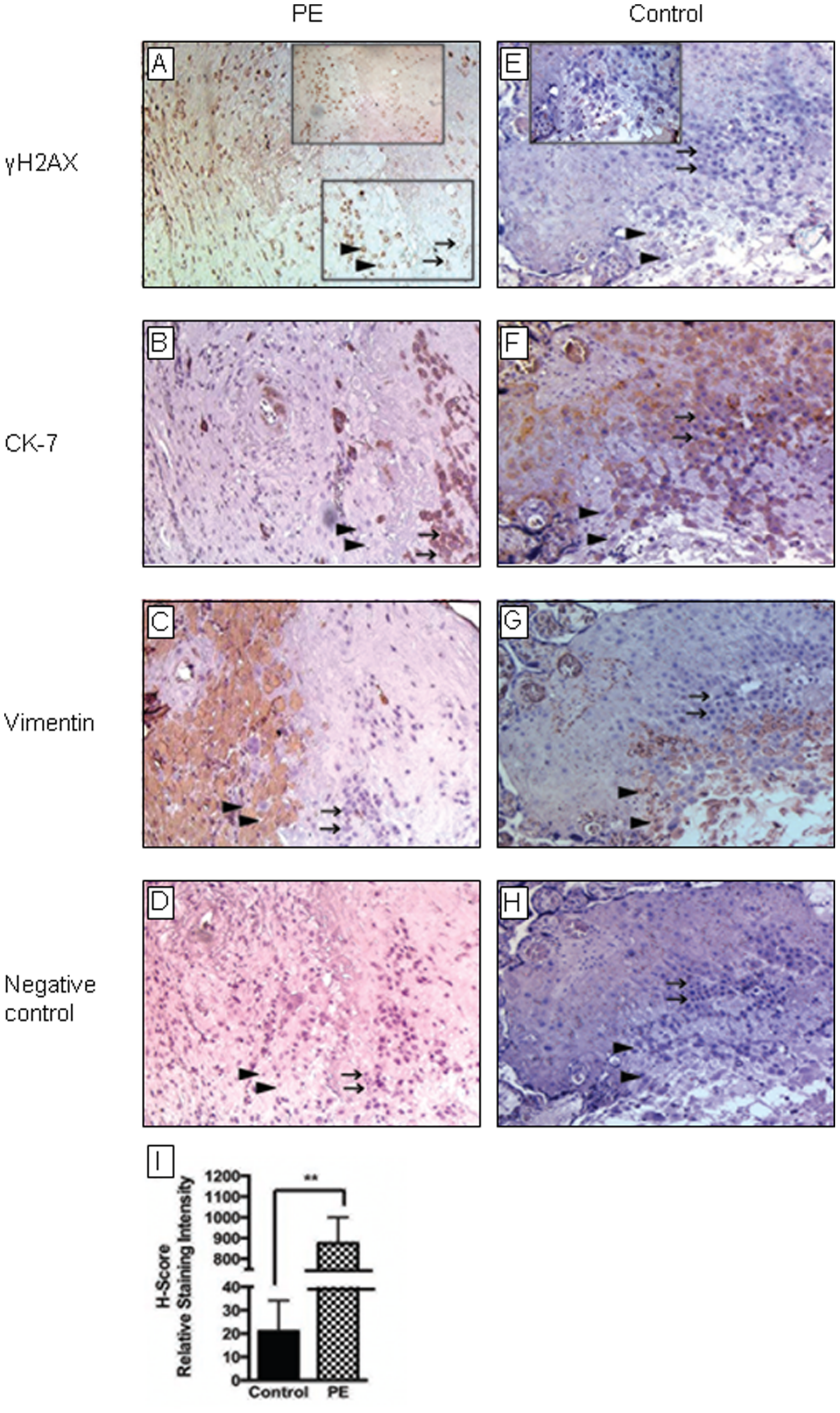
Immunohistochemical analysis of placental tissues for *in vivo* evidence of DNA damage. Placental tissues were collected from women with preeclampsia [PE] [***A–D***] and gestational age-matched normotensive controls (n = 10 for each) [***E–H***]. Tissues were prepared and labeled with antibodies against γH2AX (a biomarker of DNA damage [***A,E***]), cytokeratin 7 (CK-7) (which stains trophoblast cells, see arrows [***B,F***]), and vimentin (which stains decidual cells, see arrow heads [***C,G***]) as described in the *Materials and Methods*. Negative controls [***D,H***] included saline in place of first antibody. Representative serial tissue sections are shown. γH2AX labeling (see arrow heads in ***A***) was more intense in PE tissues than normotensive controls. The intensity of staining in decidua cells was quantified using the H-SCORE system (measured in relative staining intensity) and the data analyzed using GraphPad Prism software. Data are mean±SEM from a minimum of 3 separate experiments performed in triplicate; **P<0.001[***I***].

To determine whether oxidative DNA damage is present in the placenta and whether this is altered in the setting of PE, we performed IHC for γH2AX in placental tissues from patients with PE (n = 10) vs gestational age-matched normotensive controls (n = 10). Results demonstrate that placentas from PE patients have significantly higher expression of γH2AX vs normotensive controls (H-SCORE: 875.1±73.9 vs 21.1±7.8; mean±SEM; *P*<0.001) (Fig. 1). Indeed, while low levels of γH2AX immunoreactivity was detected in control tissues (Fig. 1A–1D), high levels were evident in PE placentas and appeared to be localized to the maternal cells of the decidua, which also stained positive for vimentin (Fig. 1C and 1G). In contrast, γH2AX immunostaining was very low in cytokeratin 7-positive trophoblast cells in both cases and controls (Fig. 1B and 1F).

### γH2AX Foci Formation in CTs and DSCS *in vitro*

**Figure 2:**
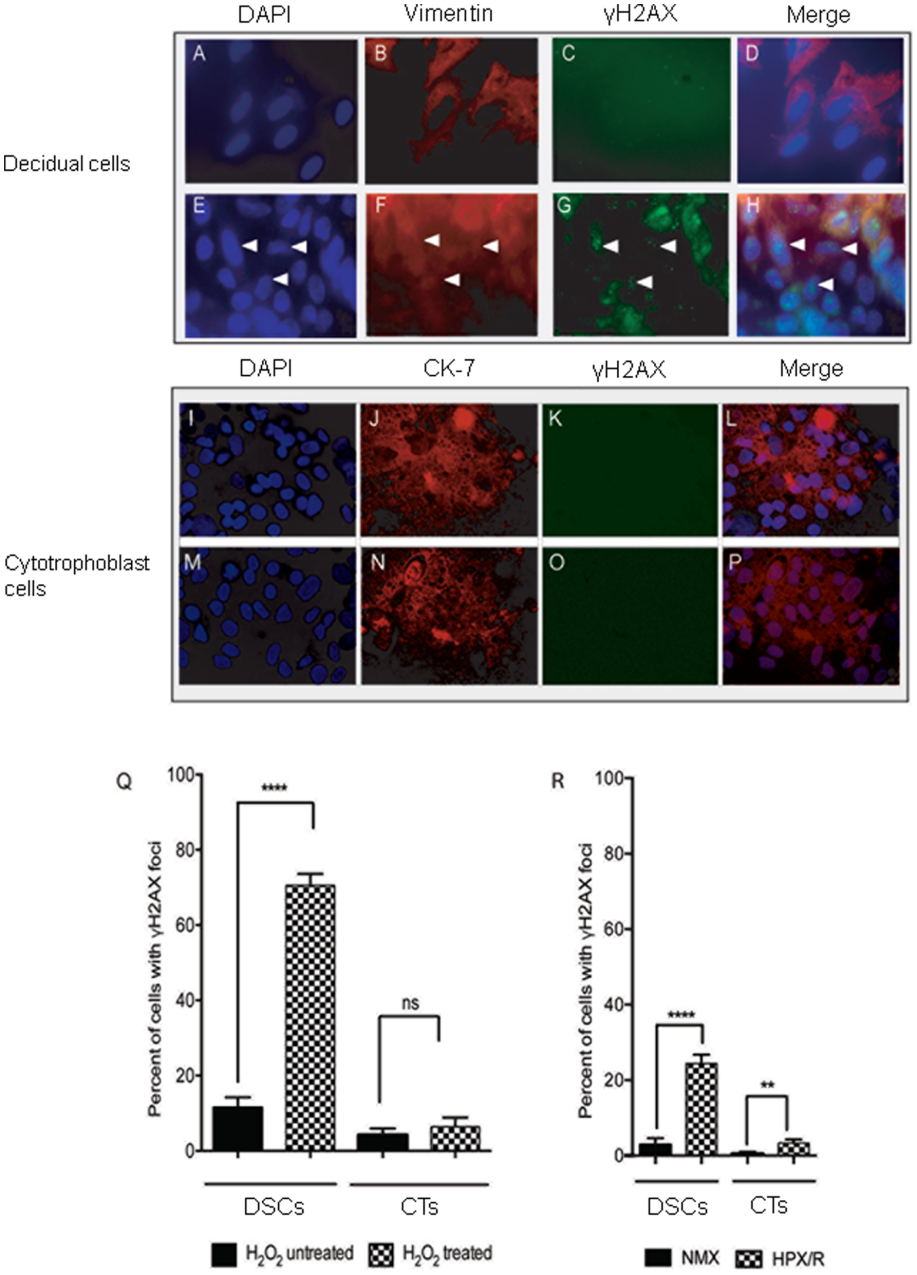
γ-H2AX focus formation in cultured decidual and cytotrophoblast cells *in vitro*. Term decidual stromal cells (DSCs) [***A–H***] were isolated, purified, and cultured without [***A–D***] or with [***E–H***] H_2_O_2_ 100 µM for 1 h to generate excess ROS. Thereafter, cells were fixed and stained with DAPI (to identify cell nuclei [***A,E***]), vimentin (to identify decidual cells [***B,F***]), and γH2AX (a biomarker of DNA damage [***C,G***]). Representative immunocytochemical images are shown. In each case, a merged image of the individual DAPI, vimentin, and γH2AX images was generated by computer analysis [***D,H***]. Identical experiments were carried out with term cytotrophoblast cells (CTs) [***I–P***], but using cytokeratin 7 (CK-7) (which stains trophoblast cells [***J,N***]) in place of vimentin. All images were taken using a Zeiss confocal microscope at 63× magnification. Quantification of γH2AX staining was based on the number of positive cells for γH2AX foci in the treated vs untreated groups (see arrows). Many more DSCs stained positive for γH2AX foci after as compared with before H_2_O_2_ treatment (70.6% [228/323 cells] vs 11.6% [147/1267 cells], respectively; *P*<0.0001), while CTs showed low foci formation with or without H_2_O_2_ treatment (6.0% [108/1800] vs 4.0% [142/3550], respectively; *P* = 0.319) [***Q***]. Similarly, significantly more DSCs stained positive for γH2AX foci when cultured under HPX/R vs NMX conditions (24.4% vs 2.9%, respectively; *P*<0.0001), whereas the response in CTs was less dramatic (3.3% vs 0.1%, respectively; *P*<0.001) **[R]**. All data were analyzed using GraphPad Prism software. Values are expressed in mean±SEM percent of cells with γH2AX foci from a minimum of 3 separate experiments performed in triplicate. **P<0.001, ****P<0.0001, ns = non-significant.

**Figure 3:**
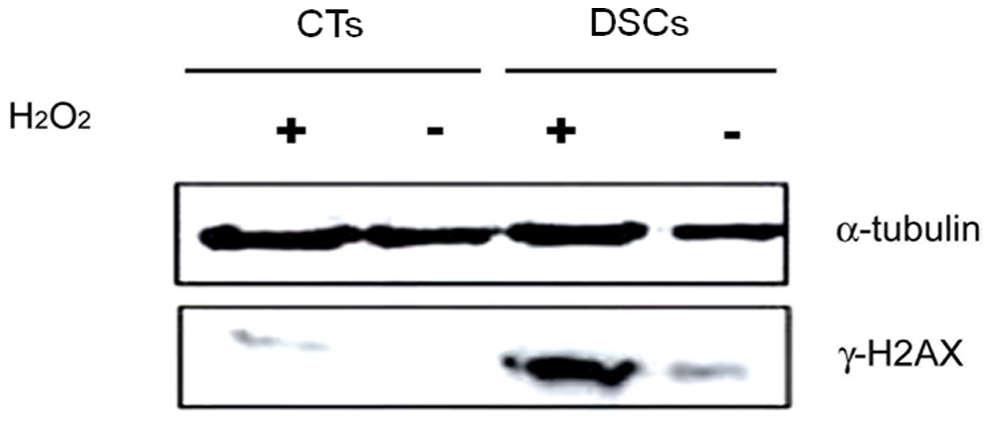
Western blot analysis. To investigate the effect of H_2_O_2_ treatment on γH2AX protein expression, Western blot analysis was performed on protein extracted from decidual stromal cells (DSCs) and cytotrophoblast cells (CTs) cultured with or without H_2_O_2_ 100 µM for 1 h as described in the *Materials and Methods*. Representative Western blots are shown, including α-tubulin control.

Phosphorylation of the histone protein H2AX in response to DNA damage results in the formation of discrete γH2AX foci at the sites of DNA double-strand breaks [33]. In an effort to recapitulate our *in vivo* placental findings, we exposed DSCs and CTs to either 100 µM H_2_O_2_ for 1 h (to generate excess ROS) or HPX/R as described, then fixed the cells and stained them with anti-γH2AX antibody. Results showed a significant increase in the number of cells staining positive for γH2AX when DSCs cells were treated as opposed to untreated with H_2_O_2_ (70.6% vs 11.6%, respectively; *P*<0.0001) (Fig. 2A–2H and Fig. 2Q). In contrast, γH2AX focus formation was low in CTs regardless of whether or not the cells were treated with H_2_O_2_ (6.0% vs 4.0%, respectively; *P* = 0.319) (Fig. 2I–2P and Fig. 2Q). To better understand the upstream events that may lead to excess ROS production and DNA damage at the fetal-maternal interface, DSCs and CTs were treated under conditions of HPX/R or NMX as described. The percentage of cells showing γH2AX foci were significantly increased in both DSCs and CTs following HPX/R, but the effect was far more dramatic in DSCs (24.4% vs 2.9%, respectively; *P*<0.0001) than in CTs (3.3% vs 0.1%, respectively; *P*<0.001*).* Western blot analysis confirmed an increase in γH2AX protein expression (approximately 2- to 3-fold) following H_2_O_2_ treatment for 1 h in DSCs, but not CTs (Fig. 3).

### Effect of Oxidative Stress on DNA AP Sites in DSCs and CTs *in vitro*

**Figure 4:**
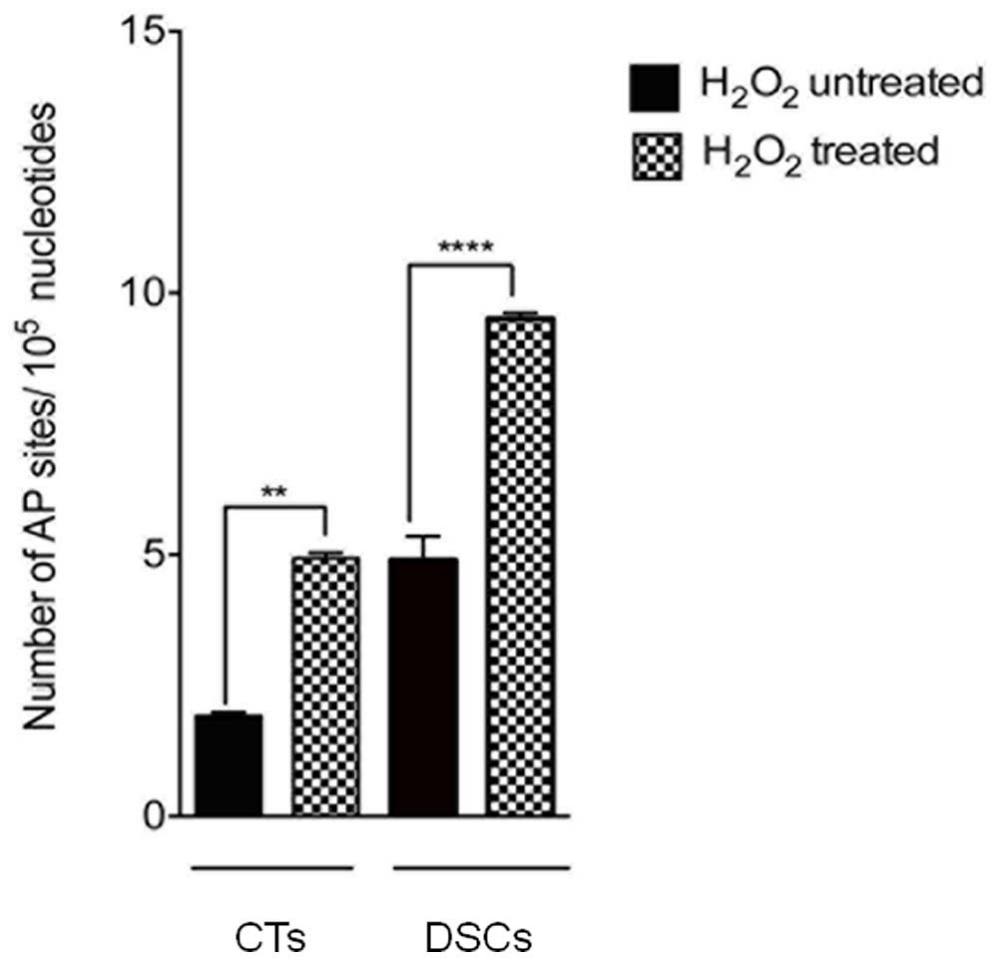
DNA apurinic/apyrimidinic (AP) sites as a measure of DNA damage in cultured decidual and cytotrophoblast cells *in vitro*. Term decidual stromal cells (DSCs) and cytotrophoblast cells (CTs) were isolated, purified, and cultured with or without H_2_O_2_ 100 µM for 1 h as described in the *Materials and Methods*. The effect of H_2_O_2_ treatment on the number of DNA AP sites was estimated using a commercial ELISA-like assay that utilizes an aldehyde reactive probe (ARP) (Abcam, Cambridge, MA). Results are expressed as fold change in the number of DNA AP sites per 10^5^ nucleotides. **P<0.001, ****P<0.0001.

To determine the amount of AP sites, genomic DNA was isolated from DSCs and CTs treated with or without 100 µM H_2_O_2_ for 1 h as described using a method previously shown not to introduce additional AP sites [30]–[32]. In response to H_2_O_2_ treatment, the number of DNA AP sites increased two-fold in both DSCs (4.7 vs 9.4 AP sites/10^5^ nucleotides, respectively; *P*<0.0001) and CTs (1.8 vs 4.9 AP sites/10^5^ nucleotides, respectively; *P*<0.001) (Fig. 4). Interestingly, the basal number of DNA AP sites was three-fold higher in DSCs than in CTs (4.7 vs 1.8 AP sites/10^5^ nucleotides, respectively; *P*<0.001). Therefore, while BER intermediates are generated on both sides of the fetal-maternal interface in response to oxidative stress, they appear to ‘accumulate more rapidly on the maternal side, which may lead to more DNA damage in the decidua.

## Discussion

The integrity of the fetal-maternal interface is critical for the survival of the conceptus. There is substantial evidence to suggest that failure of the trophoblast to adequately invade the maternal tissues of the uterus and remodel the maternal vasculature in the early second trimester leads to placental dysfunction and PE [34]. High oxygen tension at the fetal-maternal interface may lead to excessive ROS generation, which may, in turn, lead to DNA damage and interfere with trophoblast invasion and placentation. In this study, we demonstrate that DNA double-strand breaks (as evidenced by γH2AX staining) is significantly more common in the placentas of women with PE compared with gestational age-matched normotensive controls, and this increase appears to be localized *in vivo* to the cells of the maternal decidua and not the trophoblast (Fig. 1). Consistent with this observation, we further demonstrate that γH2AX protein expression is increased in cultured DSCs (but not CTs) in response to treatment with H_2_O_2_ to generate excess ROS (Fig. 2 and 3).

The phosphorylated form of the histone protein H2AX (γH2AX) is a sensitive marker of DNA double-strand breaks and repair. Prior studies in different model systems have shown that γH2AX can be visualized by immunocytochemistry of cell nuclei and chromosomes [35], [36]. We confirm that this is true also of the placenta, and we further demonstrate that the DNA damage is not randomly distributed among different placental cell population, but appears to be limited to the decidual cells. An up-regulation of DNA damage-related genes (especially ribonucleotide reductase 2) within the decidua has been shown, in a mouse model, to promote uterine cell proliferation and decidualization in early pregnancy [37]. Whether this is true also in human pregnancy is not known, although several studies have suggested that the process of decidualization in humans confers protection against oxidative stress-induced cell damage [38] and represses signal transduction pathways that promote oxidative stress-mediated cell death [39]. Regardless, excessive DNA damage at the fetal-maternal interface is likely to be pathogenic.

Although both DSCs and CTs retain the ability to generate DNA AP sites (a marker of ongoing DNA damage and repair) in response to 1 h stimulation with excess ROS, the number of DNA AP sites under both basal and stimulated conditions was three-fold higher in DSCs than CTs (Fig. 4). Why is the DNA damage/repair response more robust in DSCs? Alternatively, why is it that CTs are more resistant to γH2AX focus formation at the site of oxidative DNA damage? It may indicate, as has been demonstrated for other tissues and cells types [40], [41], that DNA BER capabilities are not equivalent between the two cell populations. Since the DNA intermediates in the BER repair pathway are themselves cytotoxic and can lead to genomic instability, a complex series of steps exists within most cells to hand off these toxic intermediates from one enzyme to the next in a coordinated, sequential fashion so that the intermediates are sequestered and DNA damage is limited [42]. One explanation is that CTs may be able to achieve this coordinated sequence of events more effectively than DSCs thereby limiting DNA damage. In this way and consistent with our hypothesis, CTs may be selectively resistant to DNA damage in an effort to protect the fetus.

In summary, the current study demonstrates that oxidative stress-induced DNA damage and repair is present in higher amounts in the placentas of women with PE vs gestational age-matched normotensive controls, and appears to be clustered in the genomic DNA of DSCs, but not CTs. These *in vivo* findings could be recapitulated *in vitro* using conditions of excess ROS and HPX/R. These data suggest that CTs may be selectively resistant to ROS-induced DNA damage. The biological importance of oxidative DNA damage remains to be determined, but it is reasonable to suggest that such DNA damage could affect the transcriptional regulation of placental genes involved in implantation and placentation and/or cell fate decisions (apoptosis) thereby contributing to the pathogenesis of PE. Further studies (including the assessment of other BER-related pathways under both basal and stimulated conditions and the possible role of genetic polymorphisms in DNA damage/repair genes) are needed to confirm these observations and to fully understand the relationship between the DNA damage/repair pathways and PE.
